# Patient Navigators in Cochlear Implant Services: A Survey of Current Practices and Utilization

**DOI:** 10.1097/ONO.0000000000000080

**Published:** 2025-11-17

**Authors:** Alejandra Ullauri, Achilles Kanaris, Cedric V. Pritchett, Alexa Velez, Kevin Yizhe Zhan

**Affiliations:** 1 Audiology En Español, Chicago, Illinois; 2 Department of Otolaryngology-Head & Neck Surgery, Division of Otology & Neurotology, Northwestern Medicine, Chicago, Illinois; 3 Division of Otolaryngology, Nemours Children’s Health, Orlando, Florida

**Keywords:** Cochlear implants, Health equity, Healthcare access, Hearing care access, Patient navigators

## Abstract

**Objective:**

To understand current patient navigator (PN) use in cochlear implant (CI) programs and assess the perceived value of PNs in this context.

**Design:**

A cross-sectional online survey was administered via Qualtrics-XM between February’24 and January’25.

**Setting:**

Survey was distributed to CI professionals via the American Cochlear Implant Alliance’s email list and social media.

**Main Outcome Measures:**

Demographics and PN use were summarized using descriptive statistics, and perceived PN value using a Likert scale from 1—not useful at all to 5—extremely useful. Barriers to care were categorized using an ecological framework.

**Results:**

In total, 84 respondents completed the survey anonymously. Most were CI audiologists (n = 58, 69%), from urban (n = 57, 67.9%) and academic clinical settings (n = 41, 48.8%). Most reported not having someone fulfilling a PN role (n = 59, 70.2%). Only 9.5% (8) had a full-time PN, and 20.2% (17) had part-time PNs. Common PN duties included: navigating financial barriers, coordinating multi-specialty visits, addressing logistical issues (eg, transportation and childcare), and patient-tracking. Four PN responsibilities were rated “extremely useful”: addressing emotional barriers, resolving logistical challenges, tracking patients, and coordinating multi-specialty visits. Totally, 251 open-ended responses spotlighted organizational barriers to care.

**Conclusions:**

PNs are uncommon in CI programs but are seen as highly valuable for addressing emotional, logistical, and systemic barriers. We offer a definition for the PN role to facilitate models of integration. As CI programs embrace both the existing disparities to care and the overall underutilization of CIs, integrating PNs into CI teams merits thoughtful consideration.

Patient navigators (PN) are part of a community-based service delivery model that promotes “access to timely diagnosis and treatment of chronic” conditions by eliminating barriers to care (1). They have been widely used in cancer care since the early 1990s and have played an instrumental role in increasing screening rates, access to treatment, and follow-up care. The first PN program, started by Dr. Harold Freeman in 1991 in Harlem, was established with the goal of improving breast cancer screening rates, access to early treatment, and follow-up care for women from underserved groups (1). Following the success seen in cancer care, PN roles have expanded to other areas such as primary care and ambulatory services, and are part of specific clinics that focus on caring for patients with chronic conditions such as diabetes and HIV (2–6).

Hearing loss is another example of a chronic condition—as defined by the Agency for Healthcare Research and Quality (7)—where patient navigation could play a valuable role. Access to evidence-based screening, diagnostics, and treatment options for hearing loss remains low despite considerable advances in clinical protocols, diagnostic, and hearing technology (8–11). Minorities and underserved populations are particularly less likely to access hearing care; (12) moreover, cochlear implant (CI) utilization rates are even lower in these population groups (10,13,14). Considering the nature of a chronic condition such as hearing loss, the complexity of accessing hearing care services, and the documented disparities in access to CI services, the present study examines the presence of PNs in CI programs in the United States with the main goal of understanding PN utilization and the potential value of PN incorporation in this clinical space.

## MATERIALS AND METHODS

### Survey Design

This study is an online cross-sectional, questionnaire-based survey, reviewed by the Northwestern University Institutional Review Board (IRB), granted exempt IRB status (IRB Study # STU00220858). The survey was created utilizing the web-based Qualtrics-XM Platform (Qualtrix Core XM Survey Software, 2020) (Qualtrics, Provo, UT) (15) and comprised of 18 questions. Sixteen multiple-choice questions assessed respondent and practice setting demographics, one Likert scale question assessed respondents perceived value of patient navigators in CI programs, and a final open-ended question sought to identify clinicians perceived barriers to care experienced by their patients seeking CI services. When respondents were queried if their CI program included a PN, we offered a definition of the role to allow respondents to consider whether they had an individual who met the description: Patient navigators promote access to timely diagnosis and treatment of chronic diseases by eliminating barriers. In cochlear implant programs, a patient navigator helps patients and families overcome barriers to hearing care and promote timely movement of an individual patient through a complex and often disconnected healthcare continuum. A patient navigator is most effective when they have a defined role in the team and they are able to establish one-on-one relationships with the patient or family.

Responses were anonymous. The full list of distributed survey questions is available in Supplemental Appendix 1, https://links.lww.com/ONO/A39. The survey was distributed to CI professionals through the American Cochlear Implant Alliance email list and through social media channels (Facebook, X, and LinkedIn). Data collection was open from February 2024 to January 2025.

### Survey Data Analysis

Statistical analysis was conducted utilizing the Qualtrics-XM Platform’s Stats iQ as well as with R Studio Version 2024.04.2 (R Core Team, 2024) (15,16). Both quantitative and qualitative analyses were employed. Descriptive statistics were utilized to describe patient demographic responses in the form of frequency count and percentage. 5-point Likert scale responses were coded 1–5, with 1 representing “not useful at all” and 5 representing “extremely useful.” Subgroups were analyzed with nonparametric methods. To account for multiple comparisons, the Bonferroni correction was utilized. A thematic analysis was independently performed on survey responses regarding barriers to care by 2 members of the team. These responses were first assigned codes, then independently grouped into the 5 themes in the ecological framework proposed by Neukam et al (17). Before identifying discrepancies, each member was blinded to the responses of the other to minimize bias in the final interpretation. All complete responses were included in the final analysis, except for one response originating from outside of the United States.

## RESULTS

### Survey Demographics

In total, 84 respondents who completed the survey were included in the final analysis. Demographics of survey respondents are presented in Table 1. The majority of respondents were CI audiologists (n = 58, 69%), largely from urban settings (n = 57, 67.9%) and the Southern United States (n = 41, 48.8%). The academic clinical setting was the most represented business model (n = 41, 48.8%). With regard to CI device activations per year, 0–25 CI device activations per year was the largest group (n = 24, 28.6%), followed by 100–200 device activations per year (n = 21, 25.0%). Private insurance was the most accepted form of payment (n = 81, 96.4%), followed by Medicare (n = 71, 84.5%), and Medicaid (n = 66, 78.6%) was the third most common in a multi-response survey question. Most survey respondents served both an adult and pediatric patient population (n = 50, 59.5%). The majority of respondents (n = 46, 54.7%) served a population where more than 10% of their patients spoke languages other than English. Fourteen respondents (16.7%) served a patient population where more than half of their patients spoke a primary language other than English.

**TABLE 1.** Survey respondent demographics

Characteristic	N = 84; n/N (%)
Primary role	
Cochlear implant audiologist	58/84 (69.0%)
Cochlear implant surgeon	14/84 (16.7%)
Speech-language pathologist	6/84 (7.1%)
Other	6/84 (7.1%)
CI device activations per year	
0–25	24/84 (28.6%)
100–200	21/84 (25.0%)
51–100	16/84 (19.0%)
26–50	14/84 (16.7%)
>200	9/84 (10.7%)
Practice setting	
Urban	57/84 (67.9%)
Suburban	20/84 (23.8%)
Rural	7/84 (8.3%)
Practice location	
South: DE, DC, FL, GA, MD, NC, SC, VA, WV, AL, KT, MS, TN, AR, LA, OK, TX	41/84 (48.8%)
Midwest: IN, IL, MI, OH, WI, IA, NE, KS, ND, MN, SD, MO	22/84 (26.2%)
West: AZ, CO, ID, NM, MT, UT, NV, WY, AK, CA, HI, OR, WA	13/84 (15.5%)
Northeast: CT, ME, MA, NH, RI, VT, NJ, NY, PA	8/84 (9.5%)
Business model (choose all)	
Academic	41/84 (48.8%)
Other	20/84 (23.8%)
Private practice	20/84 (23.8%)
Government-based (eg, Veterans Affairs, military base)	3/84 (3.6%)
Forms of payment (choose all)	
Private insurance	81/84 (96.4%)
Medicare	71/84 (84.5%)
Medicaid	66/84 (78.6%)
Government	55/84 (65.5%)
Other	2/84 (2.4%)
Patient population	
Both adult and pediatric	50/84 (59.5%)
Adult	21/84 (25.0%)
Pediatric	13/84 (15.5%)
Non-English primary language	
Less than 10%	38/84 (45.2%)
10–25%	19/84 (22.6%)
>50%	14/84 (16.7%)
26–50%	13/84 (15.5%)
Device manufacturers worked with	
3	67/84 (79.8%)
2	13/84 (15.5%)
1	4/84 (4.8%)

CI indicates cochlear implant.

### Patient Navigators in Cochlear Implant Programs

Table 2 depicts survey responses regarding the PN position. A majority of respondents reported not having an individual who meets our proposed definition of a PN (n = 59, 70.2%). Eight respondents (9.5%) had a full-time person fulfilling a PN role, 17 (20.2%) had a part-time PN, and of those (25), only 3 (12%) respondents had a person who had a PN title.

**TABLE 2.** Patient navigator current practices

Question and responses	n/N (%)
Do you have someone who meets the proposed patient navigator definition, even if their title is different? (N = 84)	
No, we do not have a patient navigator; multiple members of our team split this role	59/84 (70.2%)
Yes, we have a part-time patient navigator who also has other clinical or administrative responsibilities	17/84 (20.2%)
Yes, we have a full-time patient navigator	8/84 (9.5%)
Given that your clinic has a dedicated individual who serves as a PN, what is their title? (N = 25^a^)	
Other	15/25 (60.0%)
Patient coordinator	7/25 (28.0%)
Patient navigator	3/25 (12.0%)
Given that your clinic employs a dedicated PN, what specific responsibilities do they have? (N = 25^a^; multiple responses allowed)	
Patient-tracking to prevent loss of follow-up and encourage follow-through with care	21/25 (84.0%)
Facilitating multi-specialty coordinated visits to minimize trips to the clinic	20/25 (80.0%)
Addressing logistical barriers to medical care (eg, transportation, child care, etc.)	16/25 (64.0%)
Helping patients navigate financial barriers, such as lack of insurance or the inability to pay noncovered services	16/25 (64.0%)
Device ordering	13/25 (52.0%)
Connecting candidates with CI recipients and local support organizations	13/25 (52.0%)
Helping patients overcome mistrust, misinformation, or emotional barriers	12/25 (48.0%)
Coordinating CI team meetings	12/25 (48.0%)
Addressing language barriers	11/25 (44.0%)
Helping with program outreach initiatives in the community	10/25 (40.0%)
Troubleshooting device issues	9/25 (36.0%)
Screening for social determinants of health using standardized tools	5/25 (20.0%)
Other	1/25 (4.0%)
How is this dedicated PN position funded? (N = 25^a^; multiple responses allowed)	
Clinical overhead	19/25 (76.0%)
Grants	4/25 (16.0%)
Other	4/25 (16.0%)
Private funding	3/25 (12.0%)
Given that you do not have a dedicated PN, who do you perceive fills this role? (N = 59^b^; multiple responses allowed)	
CI audiologist	54/59 (91.5%)
Front desk associate	17/59 (28.8%)
Surgery scheduler	14/59 (23.7%)
Nurse	9/59 (15.3%)
Speech-language pathologist	8/59 (13.6%)
Medical assistant	8/59 (13.6%)
Audiology assistant	7/59 (11.9%)
CI patient coordinator	7/59 (11.9%)
CI surgeon	3/59 (5.1%)
Other	3/59 (5.1%)
What are the reasons why you do not have a dedicated PN? (N = 59^b^; multiple responses allowed)	
Lack of funding	33/59 (55.9%)
Institutional barriers	25/59 (42.4%)
Low clinical volume	13/59 (22.0%)
Patient navigation and coordination is adequately fulfilled by existing members of the team	9/59 (15.3%)
Unaware of the role	9/59 (15.3%)
Currently trying to hire a patient navigator	8/59 (13.6%)
Other	5/59 (8.5%)
How many hours do you spend performing what you perceive as PN duties? (N = 59^b^)	
3–6 hours per week	29/59 (49.2%)
0–3 hours per week	21/59 (35.6%)
6–9 hours per week	9/59 (15.3%)

a Respondents who reported having a dedicated individual serving as a patient navigator (n = 25) were asked these questions.

b Clinics without a dedicated patient navigator (n = 59) were asked these questions.

CI indicates cochlear implant; PN, patient navigator.

The most commonly reported roles fulfilled by those individuals in PN roles were: 1) patient tracking to prevent loss to follow-up and to encourage follow-through with care (n = 21, 84%); 2) facilitating multi-specialty coordinated visits to minimize trips to the clinic (n = 20, 80%); 3) addressing patient-facing logistical barriers to medical care (n = 16, 64%); and 4) helping patients navigate financial barriers such as lack of insurance or inability to pay for non-covered services (n = 16, 64%). These positions were primarily funded by clinical overhead (n = 19, 76.0%).

For those who did not have someone fulfilling a PN role (n = 59), it was commonly reported that CI audiologists in their practice were performing the duties that would be associated with the position (n = 54, 91.5%). The most cited reason for not having the position was a lack of funding (n = 33, 55.9%), followed by institutional barriers (n = 25, 42.4%). Twenty-nine respondents (49.2%) in this subgroup reported typically spending 3–6 hours per week on what they perceive as PN duties.

### Perceived Value of a Patient Navigator Role in Cochlear Implant Programs

**FIG. 1.**
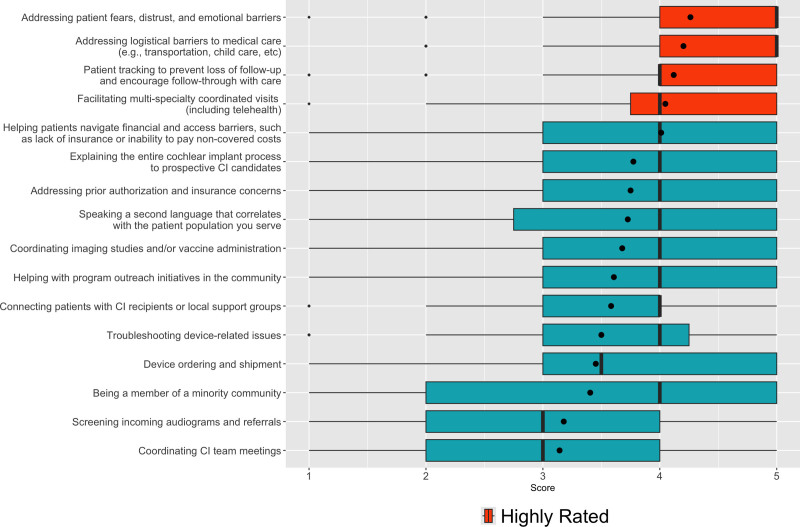
Likert Scale analysis—perception of patient navigators roles. Box plots for all Likert scale questions scores, sorted by mean score. The most valued roles are highlighted. Median and IQR are represented with dark vertical bars and box edges, respectively. Mean is represented as a black dot. Outliers are represented as diamonds.

The responses to the Likert scale question demonstrated 4 main responsibilities that respondents highly valued, based on higher median scores and narrow variability in a PN role (Fig. 1). These were 1) helping patient address fears, distrust, and emotional barriers; 2) helping patients address logistical barriers to medical care (transportation, child care, etc.); 3) tracking patients to prevent loss to follow-up; and 4) facilitating multi-specialty coordinated visits (including telehealth). Responses also highlighted roles that had lower median scores and wide variability, such as device ordering and shipment, screening incoming audiograms and referrals, and coordinating CI team meetings. Providers from programs that served >10% non-English primary language patients valued the role of speaking a second language and being a member of a minority community more than providers serving <10% non-English primary language patients (5 [1] vs 3 [2] and 4 [2] vs 3 [2] respectively, *P* < 0.001). After Bonferroni correction, no differences in valuation were observed when stratifying by program CI volume or patient population.

### Identified Barriers to Care

Respondents had an open-ended question, which gave them the possibility to enter 3 barriers to care experienced by their patients seeking CI services. A total of 252 responses were collected, and 251 were grouped into 5 themes: policy and structural, societal, organizational, interpersonal, and individual barriers. One response was excluded.

## DISCUSSION

In this study, we sought to better characterize the current utilization of PNs in CI programs in the United States. To our knowledge, this is the first study to investigate the role of PNs in CIs services. Currently, there is no consensus on the responsibilities of such a role in CI programs, nor is there a definition specific for CI services. Our study found that PNs are not commonly part of the CI team. However, CI professionals believe their role would be extremely useful in helping address specific barriers to care experienced by patients seeking CI services.

While there is no specific definition for the role or responsibilities of a PN in CI programs, our study shows that respondents view the PN’s role as one that helps patients overcome barriers to care. When respondents were asked to identify potential roles that a PN should fulfill in CI programs, they identified addressing patients fears, distrust and emotional barriers, addressing logistical barriers to care (transportation and childcare), facilitating multi-specialty coordinated visits, and tracking patients to prevent loss to follow-up as extremely useful (Fig. 1). These specific roles or responsibilities correlate with those identified by the Centers for disease control and prevention (CDC) as part of the role of a PN.

Our results show that the majority of respondents do not work with a PN in their teams (70.2%), suggesting a limited utilization of PNs in CI programs. Of those respondents who work with a PN in their CI team, their main roles or responsibilities include helping patients navigate financial barriers, such as lack of insurance or inability to pay for noncovered services, facilitating multi-specialty coordinated visits to minimize trips to the clinic, addressing logistical barriers to care (eg transportation, childcare, etc), and patient-tracking to prevent loss to follow-up and encourage follow-through with care. Furthermore, most respondents who do not work with a PN (91.5%) reported that such responsibilities are fulfilled by a CI audiologist in their team. These findings require further investigation, especially when we consider that respondents identified shortages of providers and lack of availability of appointments as one of the most common barriers to care (Table 3), and given some evidence suggesting that there is a shortage of audiologists to meet the demand for services (18,19). In light of these findings, exploring the use of PNs in CI programs might be advantageous.

**TABLE 3.** Barriers to care identified by respondents and formatted using the ecological framework proposed by Neukam et al 2024 (17)

Summary of barriers	Including	# of responses
Policy and structural barriers		
Lack of health insurance/inadequate coverage	Insurance coverage and reimbursement issues	26
Socioeconomic status		16
Budget policies restricting the number of CIs	State/government systems	1
Health literacy		11
	Total	54
Societal barriers		
Lack of awareness about CIs by public		13
Misconceptions about CIs by public		1
Education materials	Appropriate educational resources	1
Decreased reliance on professionals for education	Misinformation	2
Stigma of hearing loss	Cultural factors	3
	Total	20
Organizational barriers		
Lack of skills by professionals		
Lack of awareness from professionals		15
Inconsistent referral criteria		14
Geographic barriers of CI centers and audiology clinics	Distance, parking issues, transportation, unavailability of some services (eg, pediatric CI services)	40
Access to rehab professionals		9
Appointment availability, provider shortage		33
Lack of clinical space	Booths mostly, but also operating rooms	8
Clinical policies and management		6
Complexity of the system		3
Multiple appointments		6
Tracking loss-to follow-up		4
Language	Lack of access to testing materials in multiple languages, lack of bilingual providers	8
	Total	146
Interpersonal barriers		
Lack of social and professional support	Manufacturer support	3
Living context/marital status	Support systems (family & community)	9
Lack of community among CI users and candidates	Support with personal equipment management	3
	Total	15
Individual barriers		
Uncertainty of outcome	Expectations	4
Fear of surgery		1
Comorbidities		3
Emotional and psychosocial barriers		4
Patient motivation		1
Patient decision		3
	Total	16

CI indicates cochlear implant.

When we asked respondents to share barriers to care experienced by their patients (Table 3), they identified geographical barriers (including transportation, travel, and distance), socioeconomic status and inadequate insurance coverage, lack of availability of appointments, lack of awareness about CIs at a community and at a professional level, among others, as common barriers to care. Overall, the barriers respondents identified correlate with studies conducted in the United Kingdom, Australia, and the United States (20–22). In these studies, researchers have found that psychosocial and socioeconomic factors, time from work (21,23), travel distance to centers (22), primary language (other than English) spoken at home (20), and cost affect referral and implantation rates (20–23). The identified barriers in the literature and in our study suggest that within cochlear implant spaces, patients need individual support outside their clinical needs, supporting the notion that this type and level of support might be better provided by a PN than a clinician. This is similarly supported by other studies highlighting that the main role of a PN is to provide support and information at an individual level, and not to provide clinical care (3,4,24,25).

PNs have demonstrated—across specialties—that providing individual support and navigation assistance to patients helps programs shorten time to diagnosis, reduce waiting times, increase screening rates, and improve adherence to recommendations. It also helps patients complete the required steps towards specific treatments (eg, move through the process from dialysis to kidney transplant), start treatment earlier, and continue with follow-up care (3,26). In hearing care and cochlear implant spaces, patients—especially from minorities and underserved communities—face similar barriers and need similar assistance. We know patients from ethnic and racial minorities, as well as underserved populations, experience delays in hearing loss identification and intervention (27) and access to CI regardless of insurance coverage (14), and they also experience a lesser likelihood of implantation (28,29). Such challenges are influenced by a complex hearing care system (30,31), socioeconomic factors including insurance coverage and costs (23), lack of awareness at individual and professional level (32,33), among other factors. Considering the well-documented benefits that PN have brought to other areas of healthcare and the complexities and disparities prevalent in hearing care, it is reasonable to say that CI programs and patients have much to benefit from implementing PN in their programs.

Patient navigation has proven to be a successful strategy in improving care access for patients across several medical specialties and in helping underserved communities access care (1,2,4,16,22) Our study’s results suggest such areas where PNs can play a crucial role to improve access, and the literature highlights key characteristics of PN roles that have the potential to benefit cochlear implant candidates and programs, making it possible to scale this accepted and effective strategy laterally to another specialty. For instance, a PN can be a lay individual who possesses in-depth knowledge of the care system (6), and with formal training in medical interpretation, they can also act as interpreters and cultural ambassadors (25). They can accompany patients to appointments within the program–building trust through the process. They can assist with health literacy by reinforcing messages from providers and sharing treatment adherence tips, coordinating appointments to minimize travel time, time off work, and transportation expenses, and foster patient education to achieve self-sufficiency so patients do not have to rely on their support indefinitely (6). Additionally, PNs could also help raise awareness about cochlear implantation at a community and at an individual level by linking people to care through building relationships, explaining what to expect during appointments, touring facilities, organizing functional activities, and hosting outreach and educational events (3,6). PNs in CI programs, who are peers with lived experiences, have the potential to become cochlear implant ambassadors, raising awareness about services within different community groups.

While there is great variability with regard to the definition of a PN, the literature shows that different names are used across specialties, including PN, advocate, case manager, and community health worker, among others (2). A key distinction among the different names and possibly overlapping roles is that PNs provide emotional and informational support, not clinical care (24), and they do that by building a relationship with each individual patient and not by only coordinating care (6). Case managers, on the other hand, tend to be roles associated with healthcare professionals such as nurses and social workers, and such roles provide some degree of clinical care.

The following descriptions provide a comprehensive overview of the role of a PN or the process of patient navigation:

“…a partnership between a patient or caregiver and a navigator that seeks to proactively guide patients through the healthcare continuum to facilitate timely access to care and to foster self-management and autonomy through education and emotional support” (24).

“…a person with or without a healthcare-related background who engages with patients on an individual basis to determine barriers to accessing care or following recommended guidelines. The PN also provides information relevant to patients’ specific circumstances to facilitate self-management and access to care” (4).

“Patient navigation is defined as a patient-centered healthcare delivery model that aims to assist the individual patient in maneuvering through a complex and disconnected healthcare system to eliminate barriers to timely care” (25).

After careful consideration of the literature and analysis of our survey’s data, we propose the following definition of a patient navigator in cochlear implant programs:*A PN can be an individual with adequate and relevant training who assists patients and families in overcoming barriers to hearing care and promotes timely movement of the patient through a complex and often disconnected CI system*. It is important to consider that a PN is most effective when they have a defined role in the team and they are able to establish one-on-one relationships with the patient or family.

## LIMITATIONS

Our study has limitations that must be considered when interpreting its results. First, we had a relatively low number of respondents (84) to draw definitive conclusions. Second, the majority of those respondents were audiologists in urban academic hospitals, potentially limiting the generalizability of our results. Third, as our survey was open to all providers working in CI programs, there may be more than 1 respondent from any given CI program. Thus, our data reflects the number of respondents who work with a PN in their teams, not the number of programs that hire a PN.

## CONCLUSIONS

PNs are not commonly part of the CI team in programs in the United States. CI professionals rate the value of PNs as extremely useful, specifically when it comes to addressing patients’ fears, distrust, and emotional barriers to care, addressing logistical barriers to care (transportation, childcare, etc), facilitating multi-specialty coordinated visits, and helping track patients to prevent loss to follow-up. Considering documented healthcare disparities in access to CI services, there is a need for further research on this topic and for considering the inclusion of PNs to CI programs. To facilitate future research and role creation, we propose a definition for the role of PNs in CI programs.

## FUNDING SOURCES

None declared.

## CONFLICT OF INTEREST

AU has a consulting agreement with Advanced Bionics LLC. CVP had a consulting contract with Advanced Bionics, LLC that ended in October 2024. KYZ has consulting agreements with Advanced Bionics LLC and Cochlear Ltd. For the remaining authors, none were declared.

## Supplementary Material


