# Supplementary material for: Patient Navigators in Cochlear Implant Services: A Survey of Current Practices and Utilization
Source: Otol Neurotol Open. 2025 Nov 17;5(4):e080. doi: 10.1097/ONO.0000000000000080 (PMC12737863; doi:10.1097/ONO.0000000000000080)
Supplement: Supplementary file 1 [file ono-5-e080-s001.pdf]

Patient Navigator Survey

Start of Block: Introduction

On behalf of researchers at Northwestern University, Audiology En Español, and Nemours Children's Health, thank you so much for your interest in our study! It is designed is to examine the role of patient navigators in cochlear implant programs and should take no more than 5-10 minutes. Participation is optional. Your responses will be anonymous. There is minimal risk associated with participating. There is no compensation for completing the survey.
For a full consent form, please click the link here.

End of Block: Introduction

Start of Block: Demographics

What is your primary role at your cochlear implant clinic?

- ○ Cochlear Implant Audiologist
- ○ Cochlear Implant Surgeon
- ○ Speech Language Pathologist
- ○ Physical Therapist
- ○ Occupational Therapist
- ○ Nurse
- ○ Social Worker
- ○ Case Manager
- ○ Medical Assistant
- ○ Surgery Scheduler
- ○ Hearing Instrument Specialist
- ○ Audiology Assistant
- ○ Psychologist
- ○ Cochlear Implant Patient Navigator
- ○ Cochlear Implant Patient Coordinator
- ○ Front Desk Associate
- ○ Other: ____

How many cochlear implant activations does your program perform in a year?

- ○ 0-25
- ○ 26-50
- ○ 51-100
- ○ 100-200
- ○ >200

What is your practice location?

- ○ Rural
- ○ Urban
- ○ Suburban

Please select in which region your program is located?

- ○ Northeast: CT, ME, MA, NH, RI, VT, NJ, NY, PA
- ○ Midwest: IN, IL, MI, OH, WI, IA, NE, KS, ND, MN , SD, MO
- ○ South: DE, DC, FL, GA, MD, NC, SC, VA, WV, AL, KT, MS, TN, AR, LA, OK, TX
- ○ West: AZ, CO, ID, NM, MT, UT, NV, WY, AK, CA, HI, OR, WA
- ○ Other: ____

What business model(s) best describes your cochlear implant program? Check all that apply

- ☐ Private practice
- ☐ Academic
- ☐ Government-based (e.g. Veterans Affairs, military base)
- ☐ Other: ____

What form(s) of payment does your practice accept? Check all that apply

- ☐ Medicaid
- ☐ Medicare
- ☐ Private Insurance (Including advantage plans)
- ☐ Government-sponsored (e.g. military)
- ☐ Other: ____

What is your patient population?

- ○ Pediatric
- ○ Adult
- ○ Both

What percentage of your cochlear implant patients (candidates and recipients) speak a primary language other than English?

- ◯ Less than 10%
- ◯ 10-25%
- ◯ 26-50%
- ◯ >50%

How many device manufacturers do you work with?

- ◯ 1
- ◯ 2
- ◯ 3

End of Block: Demographics

Start of Block: Survey

With regard to your specific cochlear implant program, what do you perceive to be the top 3 barriers to care for your cochlear implant candidates?

- ◯ Barrier 1 ____
- ◯ Barrier 2 ____
- ◯ Barrier 3 ____

Page Break

With regard to your specific Cochlear Implant (CI) program, what is your perceived value of a dedicated CI patient navigator in these potential roles?

	Not at all useful	Slightly useful	Moderately useful	Very useful	Extremely useful
Speaking a second language that correlates with the patient population you serve	○	○	○	○	○
Being a member of a minority community	○	○	○	○	○
Addressing patient fears, distrust, and emotional barriers	○	○	○	○	○
Addressing logistical barriers to medical care (e.g., transportation, child care, etc)	○	○	○	○	○
Helping patients navigate financial barriers, such as lack of insurance or inability to pay non-covered costs	○	○	○	○	○
Helping with program outreach initiatives in the community	○	○	○	○	○
Screening incoming audiograms and referrals	○	○	○	○	○

Explaining the entire CI process to prospective CI candidates	○	○	○	○	○
Facilitating multi-specialty coordinated visits (including telehealth)	○	○	○	○	○
Coordinating imaging studies and/or vaccine administration	○	○	○	○	○
Patient tracking to prevent loss of follow-up and encourage follow-through with care	○	○	○	○	○
Addressing prior authorization and insurance concerns	○	○	○	○	○
Device ordering and shipment	○	○	○	○	○
Troubleshooting device-related issues	○	○	○	○	○
Connecting patients with CI recipients or local support groups	○	○	○	○	○
Coordinating CI team meetings	○	○	○	○	○

Page Break

Proposed definition: Patient navigators promote access to timely diagnosis and treatment of chronic diseases by eliminating barriers. In cochlear implant programs, a patient navigator helps patients and families overcome barriers to hearing care and promote timely movement of an individual patient through a complex and often disconnected healthcare continuum. A patient navigator is most effective when they have a defined role in the team and they are able to establish one-on-one relationships with the patient or family.1   Given this definition, do you currently have a dedicated individual who serves as a patient navigator in your clinic, even if their title is different?

○ Yes, we have a full-time patient navigator

○ Yes, we have a part-time patient navigator who also has other clinical or administrative responsibilities

○ No, we do no not have a patient navigator, multiple members of our team split this role

○ Unsure

Page Break

Display this question:

If Proposed definition: Patient navigators promote access to timely diagnosis and treatment of chron... = Yes, we have a full-time patient navigator

Or Proposed definition: Patient navigators promote access to timely diagnosis and treatment of chron... = Yes, we have a part-time patient navigator who also has other clinical or administrative responsibilities

Given that your clinic has a dedicated individual who serves as a patient navigator, what is their official title?

- ◯ Patient Navigator
- ◯ Patient Coordinator
- ◯ Other: ____

Display this question:

If Proposed definition: Patient navigators promote access to timely diagnosis and treatment of chron... = Yes, we have a full-time patient navigator

Or Proposed definition: Patient navigators promote access to timely diagnosis and treatment of chron... = Yes, we have a part-time patient navigator who also has other clinical or administrative responsibilities

Given that your clinic employs a dedicated patient navigator, what specific responsibilities do they have in your program? Check all that apply

- ▢ Screening for social determinants of health using standardized tools
- ▢ Addressing language barriers
- ▢ Helping patients overcome mistrust, misinformation, and emotional barriers
- ▢ Addressing logistical barriers to medical care (e.g., transportation, child care, etc)
- ▢ Helping patients navigate financial barriers, such as lack of insurance or inability to pay non-covered services
- ▢ Helping with program outreach initiatives in the community
- ▢ Facilitating multi-specialty coordinated visits to minimize trips to the clinic
- ▢ Patient tracking to prevent loss of follow-up and encourage follow-through with care
- ▢ Device ordering
- ▢ Troubleshooting device issues
- ▢ Connecting candidates with CI recipients and local support organizations
- ▢ Coordinating CI team meetings
- ▢ Other: ____

Display this question:

If Proposed definition: Patient navigators promote access to timely diagnosis and treatment of chron... = Yes, we have a full-time patient navigator

Or Proposed definition: Patient navigators promote access to timely diagnosis and treatment of chron... = Yes, we have a part-time patient navigator who also has other clinical or administrative responsibilities

How does your Cochlear Implant (CI) program fund this position? Check all that apply

- ☐ Grants
- ☐ Private funding
- ☐ Clinical overhead
- ☐ Other: ____

Page Break

Display this question:

If Proposed definition: Patient navigators promote access to timely diagnosis and treatment of chron... = No, we do no not have a patient navigator, multiple members of our team split this role

Or Proposed definition: Patient navigators promote access to timely diagnosis and treatment of chron... = Unsure

Given that you do not currently have a dedicated CI Patient Navigator, who currently in your clinic do you perceive to be filling in the majority of this role? Check all that apply

- ☐ Cochlear Implant Audiologist
- ☐ Cochlear Implant Surgeon
- ☐ Speech Language Pathologist
- ☐ Physical Therapist
- ☐ Occupational Therapist
- ☐ Nurse
- ☐ Social Worker
- ☐ Case Manager
- ☐ Medical Assistant
- ☐ Surgery Scheduler
- ☐ Hearing Instrument Specialist
- ☐ Psychologist
- ☐ Audiology Assistant
- ☐ Front Desk Associate
- ☐ Cochlear Implant Patient Coordinator
- ☐ Other: ____

Display this question:

If Proposed definition: Patient navigators promote access to timely diagnosis and treatment of chron... = No, we do no not have a patient navigator, multiple members of our team split this role

Or Proposed definition: Patient navigators promote access to timely diagnosis and treatment of chron... = Unsure

Given that you do not currently have a dedicated CI Patient Navigator in your practice, what is/are the reason(s)? Check all that apply

- ☐ Low clinical volume
- ☐ Lack of funding
- ☐ Patient navigation and coordination is adequately fulfilled by existing members of the team
- ☐ Unaware of the role
- ☐ Institutional barriers
- ☐ Currently trying to hire a patient navigator
- ☐ Other: ____

Display this question:

If Proposed definition: Patient navigators promote access to timely diagnosis and treatment of chron... = No, we do no not have a patient navigator, multiple members of our team split this role

Or Proposed definition: Patient navigators promote access to timely diagnosis and treatment of chron... = Unsure

Outside of your standard clinical role as a ${Role/ChoiceGroup/SelectedChoicesTextEntry}, how many hours per week do you perform what you perceive to be patient navigation or care coordination roles?

- 0-3 hours
- 3-6 hours
- 6-9 hours
- 9+ hours

End of Block: Survey
